# Single-cell protein-mRNA correlation analysis enabled by multiplexed dual-analyte co-detection

**DOI:** 10.1038/s41598-017-03057-5

**Published:** 2017-06-05

**Authors:** Haibiao Gong, Xiaohui Wang, Benjamin Liu, Stephane Boutet, Ilona Holcomb, Gajalakshmi Dakshinamoorthy, Aik Ooi, Chad Sanada, Gang Sun, Ramesh Ramakrishnan

**Affiliations:** Fluidigm Corporation, South San Francisco, CA, 94080 USA

## Abstract

We have investigated the correlation between proteins and mRNAs in single cells employing an integrated workflow for dual-analyte co-detection. This is achieved by combining the oligo extension reaction (OER), which converts protein levels to DNA levels, with reverse transcription for mRNA detection. Unsupervised gene expression profiling analysis, including principal component analysis and hierarchical clustering, revealed different aspects of the protein-mRNA relationship. Violin plot analysis showed that some genes exhibited similar distribution patterns for proteins and mRNAs. We also demonstrate that cells can be separated into subpopulations based on their protein-mRNA expression profiles, and that different subpopulations have distinct correlation coefficient values. Our results demonstrated that integrated investigations of mRNA and protein levels in single cells allows comprehensive analysis not attainable at bulk levels.

## Introduction

The ability to measure gene expression at single-cell resolution is of increasing importance, in particular for cancer genomics, where cell-to-cell heterogeneity is an intrinsic feature^1^. Traditional single-cell gene expression analysis involves techniques such as fluorescent *in situ* hybridization, achieved by direct observation under a microscope, or by flow cytometry sorting of cells stained with fluorescence-labeled antibodies or nucleic acids^2^. However, to obtain a more comprehensive understanding of the complex molecular networks in the living cell, a highly multiplexed approach is necessary. To this end, techniques such as qPCR^3, 4^ and next-generation sequencing^5^ for mRNA analysis, and mass cytometry^6^ and oligonucleotide extension reaction (OER, also known as proximity extension assay)^7–10^ for protein detection, have been applied in single-cell gene expression analysis.

Integrated co-detection of proteins and mRNAs from the same cell has the potential to not only reveal the correlation between these two classes of biologically important molecules, but to also help understand the mechanisms of gene regulation, at both the transcriptional and translational levels. However, this task is challenging due to the following reasons: (1) Most techniques for protein and mRNA detection are incompatible; (2) Because single cells contain very small amounts of protein and mRNA materials, high sensitivity is required. (3) Non-specific cross-reactivity of antibodies and the spectral overlap of fluorescent reporters complicate multiplexed protein detection. Nevertheless, progress has been made in this field in recent years. Simultaneous detection of proteins and mRNAs in fixed cells has been achieved by using a fluorescence-based protocol^11^ or by using mass cytometry for high multiplicity^12^. In this strategy, the reactions for protein and mRNA detection are performed sequentially. Alternative approaches have involved the splitting of cell lysates into separate reactions for protein and mRNA detection^13–15^, which could lead to a loss of sensitivity for rare transcripts and proteins. To simplify workflow and reduce variability, a method that combines the protein and mRNA detection in a single reaction has been reported^16^, although a systematic analysis of the interrelationship between protein and RNA expression remains unexplored.

## Results

**Figure 1:**
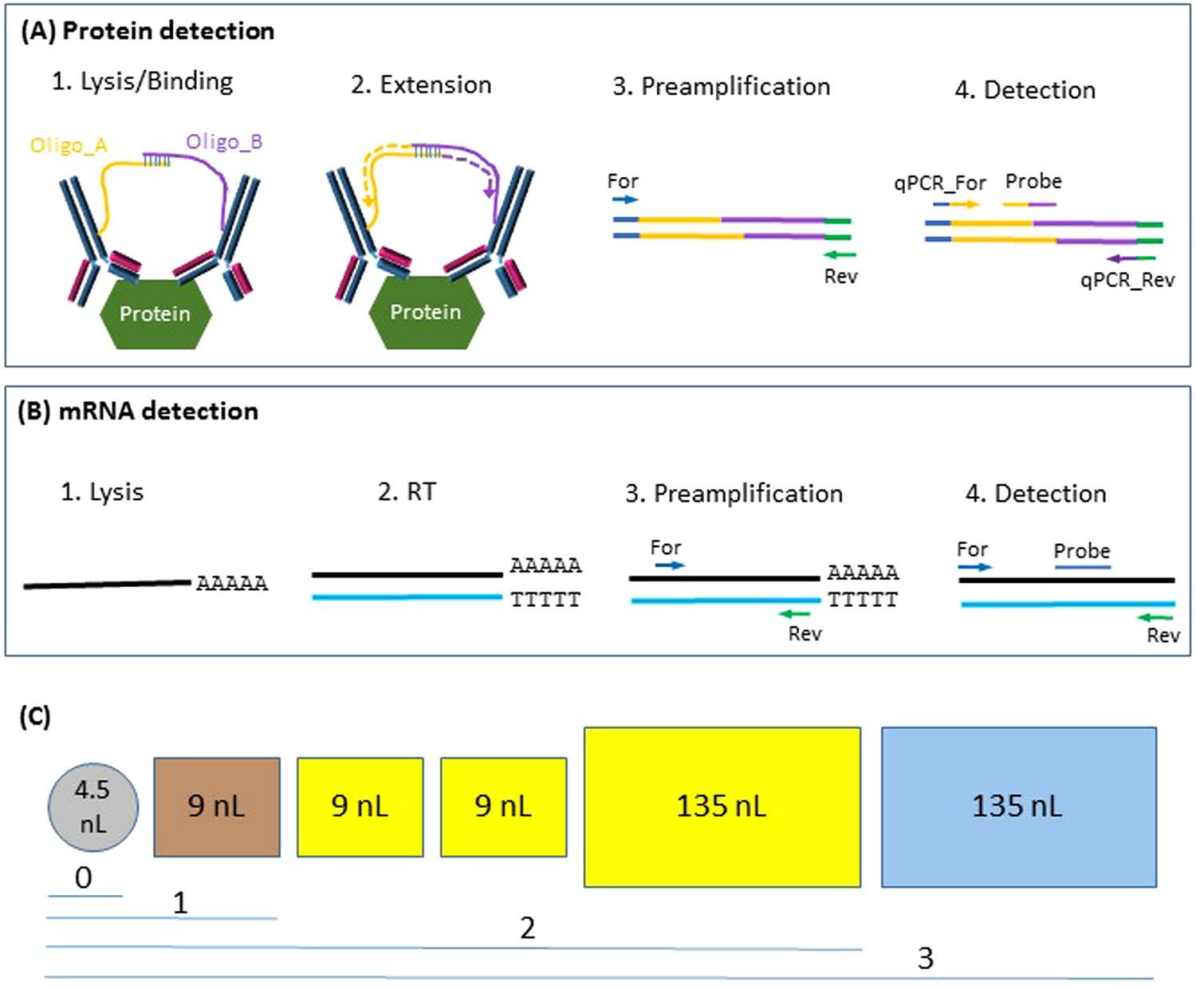
Simultaneous detection of proteins and mRNAs in single cells. (**A**) Schematic representation of the protein detection procedure (Adapted from reference^7^ with permission). (**B**) Schematic representation of the mRNA detection procedure. Steps 1–3 of protein detection and mRNA detection are in the same reactions, which are conducted in C1. For mRNA detection, both oligo(dT) and random hexamers were used to prime cDNA synthesis. Only oligo(dT) is shown for simplicity. (C) C1 chambers used for cell capture (labeled as “0”) and reaction steps 1–3.

Here we combine the integrated multiplex co-detection of proteins and mRNAs with comprehensive bioinformatics analysis to investigate their correlation in single cells. This workflow couples OER for protein detection with reverse transcription (RT) for mRNA detection and includes four steps (Fig. 1A,B), which are similar to the recently published method^16^. In the first step, cells are lysed to release proteins and mRNAs. Protein detection antibody pairs are added in the lysis reaction to allow binding to their specific targets. In the second step, protein and mRNA levels are converted to DNA levels by OER and reverse transcription, respectively. The DNA molecules are preamplified in the third step and detected by qPCR in the final step. Cell capture and the first three steps are performed in the C1 system (Fig. 1C). Technical variability is minimized by performing the lysis/binding, extension/RT and preamplification steps in the same reaction mixes without physical separation.

**Figure 2:**
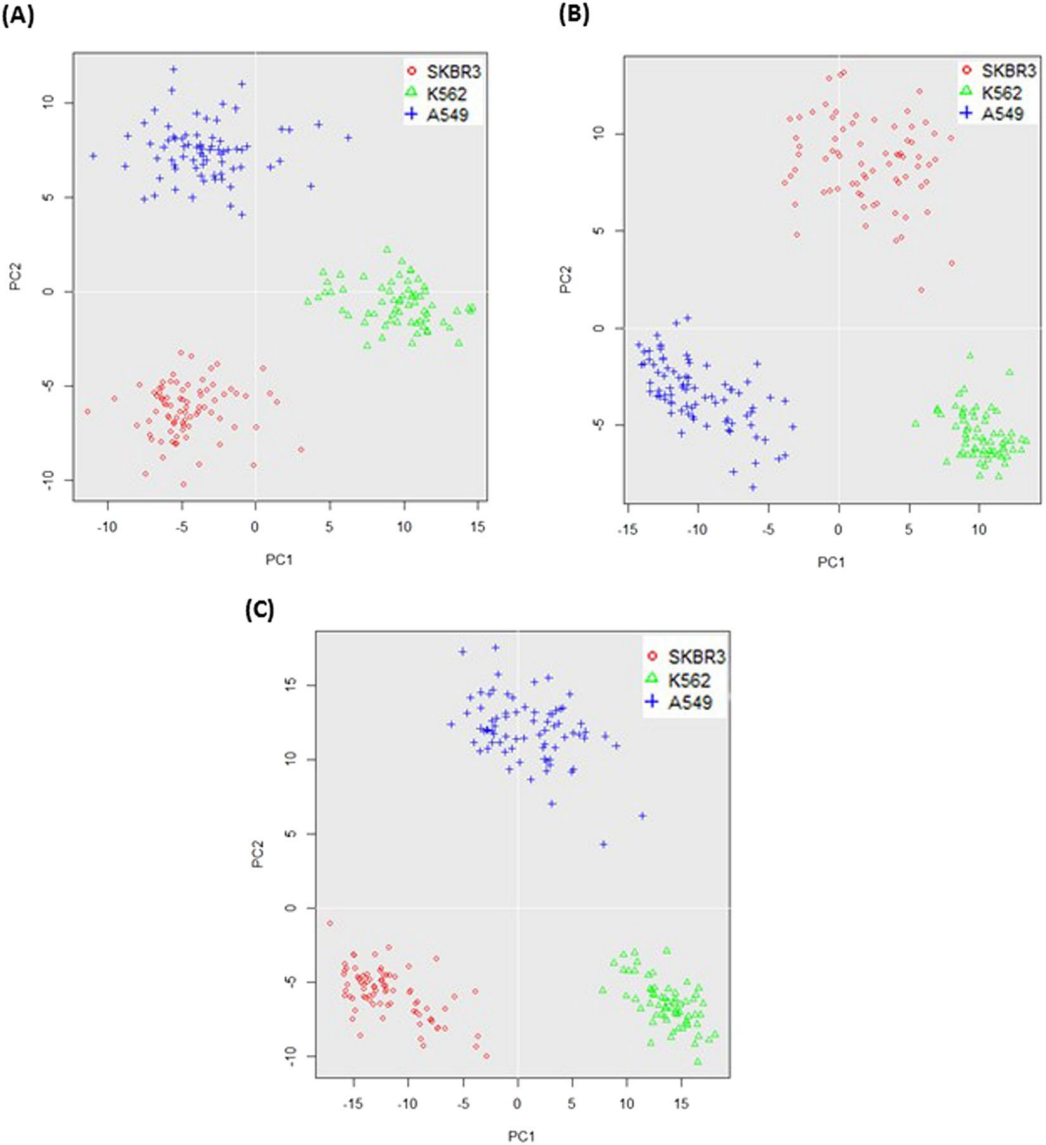
Principal component analysis (PCA) of A549, SKBR3 and K562 single cells based on either protein levels (**A**), mRNA levels (**B**), or both protein and mRNA (**C**) of 31 genes.

A panel of 84 protein assays and 40 mRNA assays were examined, and 31 of them overlap (Supplementary Table 1). The expression of proteins and mRNAs in three cell lines (A549, 74 cells; SKBR3, 81 cells; and K562, 65 cells) was analyzed. Unless otherwise noted, the 31 genes that have both protein and mRNA assays were used for analysis. Principal component analysis (PCA) using either proteins (Fig. 2A), mRNAs (Fig. 2B) or both (Fig. 2C) resulted in a clear separation of three groups, representing A549, SKBR3 and K562 cells, respectively. The top 10 genes contributing to cell differentiation were identified for both proteins and mRNAs, and eight of them overlap (Supplementary Table 2). This result implies an overall concordance of certain protein and mRNA expression in different cell lines. Hierarchical clustering analysis also demonstrated that A549, SKBR3 and K562 cells could be separated based on either proteins (Supplementary Fig. 1), mRNAs (Supplementary Fig. 2), or both (Supplementary Fig. 3). Interestingly, when both proteins and mRNAs were used for hierarchical clustering analysis, four genes (F3, NANOG, TIMP2 and CXCL8) had their protein and mRNA clustered next to each other.

**Figure 3:**
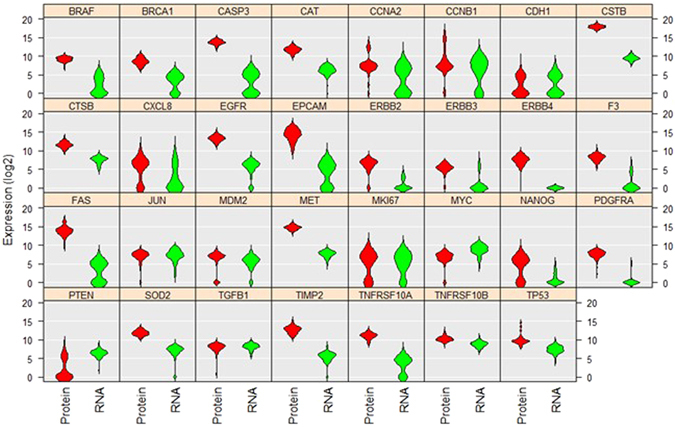
Violin plot analysis comparing the levels and distributions of 31 proteins and mRNAs in A549 single cells. The vertical position of each histogram represents the relative expression level of proteins and mRNAs (Log2Ex). A total of 74 cells were used for analysis. The separation of cells into two groups in the same histogram indicates bimodal expression pattern.

Our integrated workflow for protein and mRNA co-detection at the single-cell resolution provides a powerful tool for investigating the correlation between proteins and RNAs. First we conducted violin plot analysis to compare the expression of proteins and mRNAs in single A549 cells. Although the expression levels varied, some genes such as CAT, CDH1, CSTB, CTSB, MET, MKI67, MYC, SOD2, TIMP2, TNFRSF10B and TP53 exhibited similar distribution patterns for proteins and mRNAs (Fig. 3). Five genes (CDH1, CXCL8, MKI67, NANOG and PTEN) exhibited bimodal protein expression patterns. In contrast, 11 genes (BRAF, BRCA1, CASP3, CCNA2, CCNB1, CDH1, CXCL8, EPCAM, FAS, MKI67 and TNFRSF10A) showed bimodal mRNA expression patterns.

**Figure 4:**
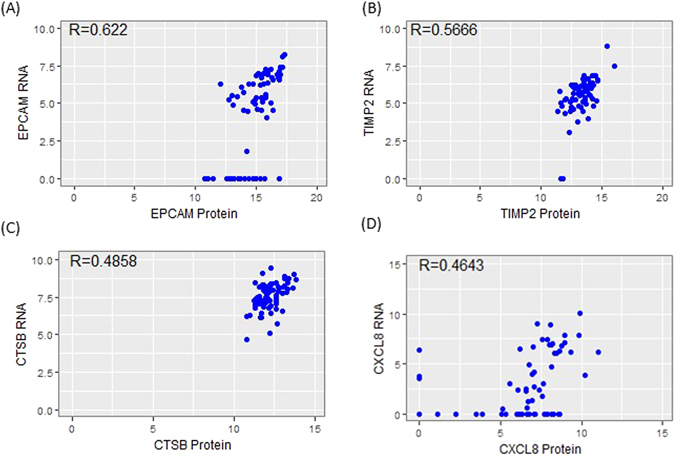
Correlation analysis between protein and mRNA levels of EPCAM (**A**), TIMP2 (**B**), CTSB (**C**) and CXCL8 (**D**).

**Figure 5:**
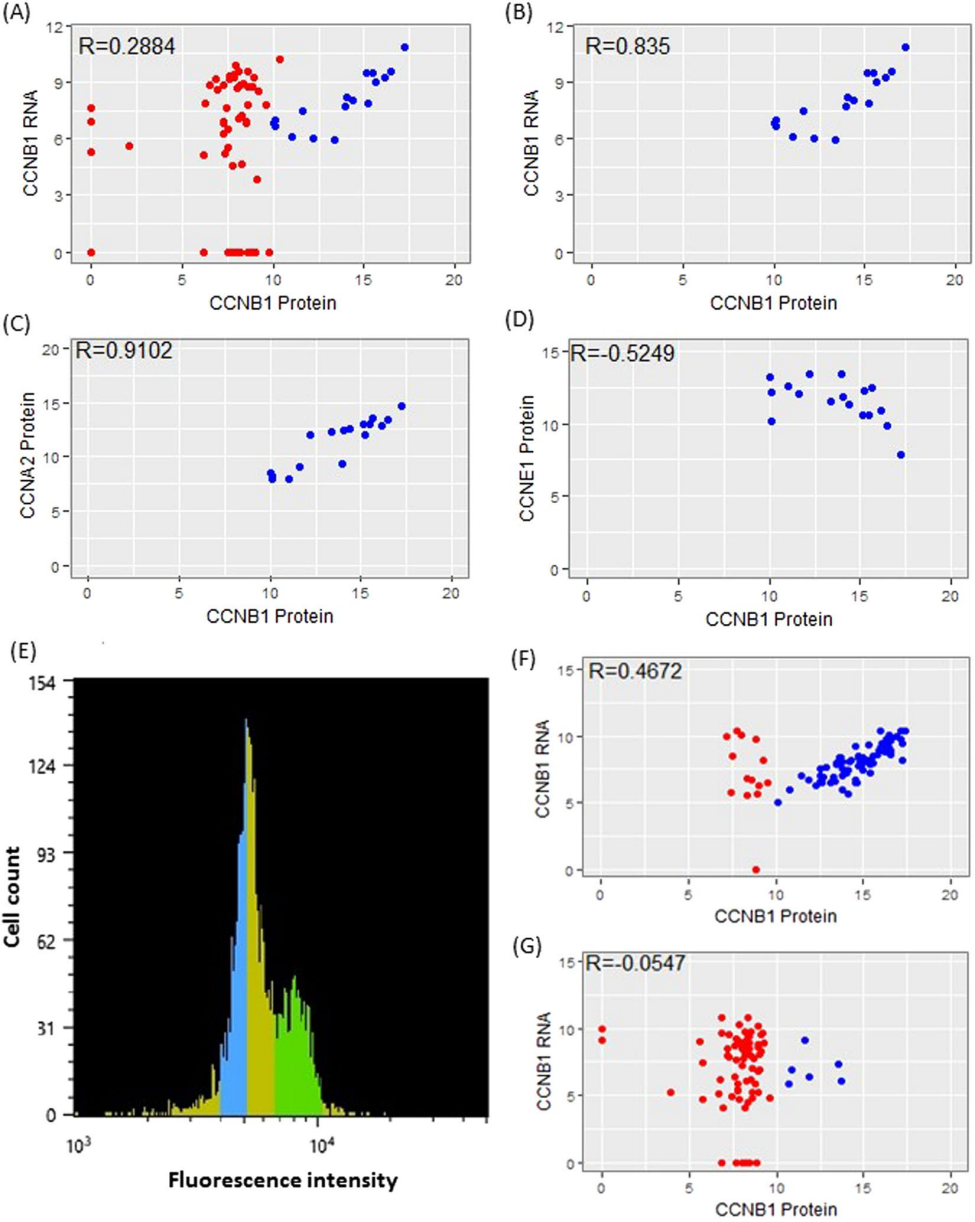
Correlation analysis of proteins and mRNAs in A549 single cells. (**A**) Correlation between CCNB1 protein and mRNA in whole population of cells. (**B**) Correlation between CCNB1 protein and RNA in the subpopulation highlighted with blue color in (**A**). (**C**) Correlation between CCNB1 protein and CCNA2 protein in the same subpopulation as in (**B**). (**D**) Correlation between CCNB1 protein and CCNE1 protein in the same subpopulation as in (**B**). (**E**) A549 cells were sorted based on DNA content. The green portion presumably represented dividing cells (S/G2/M), while the blue portion mainly represented resting cells (G0/G1). (**F**) Correlation between CCNB1 protein and RNA in A549 cells corresponding to the green fraction in (**E**). (**G**) Correlation between CCNB1 protein and RNA in A549 cells corresponding to the blue fraction in (**E**).

We next conducted scatter plot analysis between proteins and mRNAs for the same genes. To eliminate the impact of non-relevant factors (such as variation in cell volume^17^), ∆Ct values of each individual cell were normalized to the median ∆Ct of all targets for that cell^5, 18^. Results revealed varied correlation coefficient values (R) for different genes (Supplementary Fig. 4). Genes that have relatively high R values include EPCAM (R = 0.6220), TIMP2 (R = 0.5666), CTSB (R = 0.4858), and CXCL8 (R = 0.4643) (Fig. 4). Although the correlation between the CCNB1 protein and mRNA was low (R = 0.2884) in the entire population (Fig. 5A), there was a subpopulation of cells where the CCNB1 protein and mRNA levels correlated strongly (Fig. 5A, blue-colored cells). In fact, the R value within this subpopulation (subpopulation I) was 0.8350 (Fig. 5B), whereas the remaining cells (subpopulation II) had an R value of 0.0233. More interestingly, in subpopulation I, the CCNB1 protein level correlated positively with CCNA2 protein (R = 0.9102) but negatively with CCNE1 protein (R = −0.5249) (Fig. 5C,D). It is known that CCNA2 is absent or expressed at low levels in G1 and appears abundantly at the S, G2 and M phases. CCNB1 protein is also undetectable in G1 but increases dramatically in G2 and M phases. On the contrary, CCNE1 is mainly expressed during the G1/S transition^19–22^. Because the expression pattern of CCNB1, CCNA2 and CCNE1 proteins in subpopulation I matches S, G2 and M phases, this subpopulation may represent actively dividing cells. To test this hypothesis, A549 cells were sorted into two groups based on their DNA content. Group H (green-colored) had high DNA content and presumably contained mostly dividing cells (S/G2/M), while group L (blue-colored) with low DNA content contained primarily resting cells (G0/G1) (Fig. 5E). Scatter plot analysis showed that the majority of group H cells exhibited good correlation between CCNB1 protein and mRNA (Fig. 5F, blue-colored cells). When compared to unsorted cells, the subpopulation with good CCNB1 protein and mRNA correlation in group H was enriched from 23% to 84%. When this subpopulation was analyzed separately, the R value between CCNB1 protein and mRNA was 0.8211 (Supplementary Fig. 5A). CCNB1 protein also correlated positively with CCNA2 protein (R = 0.8774), and negatively with CCNE1 protein (R = −0.6164) in this subpopulation (Supplementary Fig. 5B,C). In contrast, group L contained only six dividing cells (Fig. 5G, blue-colored cells). PCA demonstrated that the resting cells from groups H and L formed one cluster and most of the dividing cells from groups H and L were also clustered together when either proteins or the combination of both proteins and mRNAs were used. It was also noted that the combination of proteins and mRNAs had a better separation (Supplementary Fig. 6A,C). However, PCA with mRNAs did not generate such a clear separation of resting and dividing cells (Supplementary Fig. 6B). This observation suggests that proteins might be more informative than mRNAs for subpopulation analysis.

Although the biological significance regarding the higher correlation between CCNB1 protein and RNA in dividing cells is not clear, this result is consistent with the observation that dynamic genes have higher protein-mRNA correlation than stable genes associated with housekeeping functions^23^. This phenomenon was also observed in SKBR3 cells. The R values between CCNB1 protein and mRNA in the whole population and subpopulation I were 0.4937 and 0.8700 (Supplementary Fig. 7A,B), respectively. Similar to the results with A549 cells, CCNB1 protein correlated positively with CCNA2 protein (R = 0.9109) but negatively with CCNE1 protein (R = −0.9505) in subpopulation I (Supplementary Fig. 7C,D).

## Discussion

Gene expression analysis at the protein and mRNA levels has been an essential tool for investigating intrinsic genomic programs and cellular responses to stimuli, leading to the construction of complex signaling pathways. Because proteins are the translational products of mRNAs, it is of great interest to study the correlation between protein and mRNA levels of the same genes. Global quantification of gene expression in bulk mouse fibroblast samples has previously revealed a significant correlation between protein and mRNA levels (R^2^ = 0.41)^24^. However, this analysis was conducted using a large group of genes, and the correlation between specific proteins and mRNAs of individual genes was not analyzed. Darmanis *et al*. studied the correlation between proteins and mRNAs of the same genes using FACS-sorted glioblastoma cells, where single cells were lysed and split for subsequent protein and mRNA analysis. For the 22 genes analyzed in that study, the overall correlation was poor^14^. It is possible that the process of sample splitting introduced variability, as well as reduced sensitivity at both protein and mRNA levels. Our approach obviates these problems by integrating protein and mRNA detection in single nanoliter-scale reactions. A systematic comparison of RNA-seq results using nanoliter and microliter reactions has demonstrated certain advantages of nanoliter reactions, such as higher accuracy and sensitivity, as well as reduced bias^5^. This may explain, at least in part, an overall better correlation between proteins and mRNAs observed in this study.

We conducted unsupervised gene expression profiling analysis, including PCA and hierarchical clustering, on protein and mRNA levels from A549, SKBR3 and K562 single cells. The results revealed different aspects of the protein-mRNA relationship although further studies are needed to better understand the biological implications of these observations. We also conducted violin plot analysis and showed that more genes exhibited bimodal mRNA expression than bimodal protein expression patterns (eleven genes for mRNA versus five genes for protein). This observation is consistent with the belief that proteins are generally more stable, while the burst-like stochastic activation of transcription causes more variation in mRNA abundance^25, 26^.

Scatter plot analysis demonstrated that a number of genes had significant correlation between protein and mRNA levels. Notably, the correlations between CCNB1 protein and mRNA levels in dividing cells are much higher when compared to the whole cell population. It is reasonable to speculate that other genes may also have tighter regulation of proteins and mRNAs in a subpopulation, or under certain conditions. More comprehensive analysis, especially studies with a higher number of genes, may help shed light on this.

In conclusion, we have developed a streamlined workflow that enables the multiplexed co-detection of proteins and mRNAs in single cells. We demonstrated that PCA, hierarchical clustering and violin plot analysis could reveal different aspects of relationship between proteins and mRNAs. Cluster plot analysis of proteins and mRNAs of the same genes showed varied R values. Interestingly, CCNB1 protein and mRNA have moderate correlation in the entire population but are tightly co-regulated in the dividing subpopulation. Integrated investigations of mRNA levels and the encoded protein levels in single cells allows comprehensive analysis not attainable at bulk levels and may reveal additional layers of complexity of gene expression regulation.

## Methods

### Chemicals and reagents

All oligonucleotides and qPCR probes were purchased from Integrated DNA Technologies (Coralville, IA). The qPCR primer and probe sequences are provided in Supplementary Table 3. Antibodies and recombinant proteins were from R&D Systems (Minneapolis, MN) unless otherwise noted. Antibody conjugation and purification were performed as described previously^10^. All other reagents were from Fluidigm (South San Francisco, CA).

### Cell culture and cell sorting

The cell lines A549, K562 and SKBR3 were obtained from the American Type Culture Collection (ATCC, Manassas, VA) and maintained in complete RPMI-1640, Iscove’s Modified Dulbecco’s, McCoy’s 5 A media, respectively. All complete media were prepared by supplementing the respective basal media with 10% fetal bovine serum (FBS).

To separate cells into dividing (S/G2/M) and resting (G0/G1) groups, A549 cells were resuspended in complete RPMI-1640 medium at a concentration of 10^6^ cells /mL and stained with Vybrant DyeCycle Green Stain (Thermo Fisher Scientific, Carlsbad, CA). The final concentration of Vybrant DyeCycle Green Stain in cell solution was 10 µM. After staining at 37 °C for 30 min, cells were sorted into separate groups based on fluorescence intensity on a LE-SH800 cell sorter (Sony Biotechnology, San Jose, CA) using 488 nm laser excitation and 525/50 nm detection filter.

### Multiplex co-detection of proteins and mRNAs in single cells

Cell capture in the C1 system was carried out as described previously^10^. In brief, the cells were washed and resuspended in Cell Preparation Buffer (C1 Protein kit) at a concentration of 200 cells/µL. The cells were then mixed with C1 Cell Suspension Reagent (C1 Protein kit) at a 3:2 ratio and loaded immediately on a C1 IFC along with 20 µL freshly prepared LIVE/DEAD staining buffer (ethidium homodimer-1 and calcein AM, Thermo Fisher Scientific). After cell loading, C1 chambers were imaged with a Leica DMI 4000B microscope in the bright field, GFP and CY3 channels for cell occupying information (either empty, live cell or dead cell) assignment.

The cell lysis/antibody binding, oligonucleotide extension/reverse transcription and preamplification steps were performed in the C1 system. All reagents were from Fluidigm unless noted otherwise. This procedure needs three reaction mixes. 1) Lysis & Ab Binding Final Mix, containing 1 µL RNAse inhibitor (Clontech, Mountain View, CA), 1 µL diluted ArrayContro RNA Spikes #4 (Thermo Fisher Scientific, 100X dilution in nuclease-free water), 8 µL C1 Ab Binding Buffer, 2 µL C1 Loading Reagent, 3 µL C1 Protein Lysis Buffer, 6 µL C1 Protein Probe Mix A, 6 µL C1 Protein Probe Mix B and 13 µL nuclease-free water; 2) Template Final Mix, containing 2 µL RNAse inhibitor, 27 µL C1 Template Master Mix, 6 µL C1 Loading Reagent and 85 µL nuclease-free water; and 3) Preamplification Final Mix, containing 3.5 µL primer cocktail (1.3 µM each) for cDNA preamplification, 1.8 µL C1 Preamp Primers for DNA template (from protein) preamplification, 35.7 µL Preamp Master Mix, 4 µL C1 Loading Reagent and 35 µL nuclease-free water. Eight µL of Lysis & Binding Final Mix, 27 µL Template Final Mix and 25 µL Preamplification Final Mix were loaded on the C1 IFC in their respective input inlets. After the program was completed, the reaction products were harvested into a 96-well plate and diluted four-fold with DNA suspension buffer for qPCR detection. The thermal conditions are as follows: 1) Lysis and Ab binding, 37 °C for 30 min; 2) Extension/reverse transcription, 40 °C for 16.5 min followed by 85 °C for 5 min; 3) Preamplification, 95 °C for 5 min followed by 18 cycles of 95 °C for 30 sec, 54 °C for 1 min and 60 °C for 1 min.

### Detection by qPCR on the Biomark HD system

The qPCR was conducted on an M96 IFC with the Biomark HD system (Fluidigm). Detection of DNA templates from proteins and mRNAs was carried out separately using their specific qPCR assays. Each qPCR assay contain 9 µM forward primer, 9 µM reverse primer and 2 µM qPCR probe. The target-specific assays (5 µL per inlet) were loaded on the assay side of the M96 IFC. Three µL of diluted C1 harvest sample was mixed with 3 µL C1 Protein Detection Master Mix (C1 Protein kit) and loaded in the M96 IFC inlets on the sample side (5 µL per inlet). After loading and mixing on an HX Controller, the M96 IFC was transferred to the Biomark HD system for PCR reaction and data acquisition.

### Data analysis

For single-cell protein detection in the C1 system, a recombinant protein of non-mammalian source was used as the OER assay control. The raw data were presented as Ct values. Ct values of all protein targets were normalized against the OER assay control. Similarly, Ct values of mRNAs were normalized against ArrayControl RNA Spikes #4. The ∆Ct values (also known as Log2Ex) were calculated using the following formula: ∆Ct = LOD – Ct. In our analysis, LOD (limit detection) was set as 24. All ∆Ct values less than zero were set as zero.

Violin plot analysis, PCA and hierarchical clustering analysis were performed using the Fluidigm Singular Analysis Toolset 3.5.2 R package. The top 10 ranked PCA genes were selected based on the maximum absolute value of each gene loading score in the first three eigenvectors (PC1, PC2 and PC3). In hierarchical clustering analysis, genes are clustered on the basis of Pearson correlation. Samples are clustered on the basis of a Euclidian distance matrix with complete linkage.

## Electronic supplementary material

Supplementary tables and figures
