# Supplementary material for: Single-cell protein-mRNA correlation analysis enabled by multiplexed dual-analyte co-detection
Source: Sci Rep. 2017 Jun 5;7:2776. doi: 10.1038/s41598-017-03057-5 (PMC5459813; doi:10.1038/s41598-017-03057-5)
Supplement: Supplementary file 1 — Supplementary tables and figures [file 41598_2017_3057_MOESM1_ESM.pdf]

Single-cell protein-mRNA correlation analysis enabled by multiplexed dual-analyte co-detection

Haibiao Gong[1,*], Xiaohui Wang[1], Benjamin Liu, Stephane Boutet, Ilona Holcomb,

Gajalakshmi Dakshinamoorthy, Aik Ooi, Chad Sanada, Gang Sun, Ramesh Ramakrishnan[*]

Supplementary table 1. List of protein and RNA assays.

Protein (84): AHR AKT1 BIRC5 BRAF BRCA1 BSG CASP3 CASP8 CAT CCNA2 CCNB1 CCND1/2 CCNE1 CD4 CD44 CDH1 CDKN2A CEACAM1 CHEK1 CSTB CTLA4 CTSB CTSD CXCL8 EGFR ENO2 EPCAM ERBB2 ERBB3 ERBB4 ESR1 F3 FAS FASLG FLT1 FOLR1 GITR ICAM1 IFNG IGF1 IL6 JNK JUN KDR KLK3 MDM2 MET MKI67 MMP1 MMP9 MUC1 MYC NANOG NDRG1 NFE2L2 NFKB1 NOTCH1 PD1 PDGFRA PDL1 PDL2 PGR POU5F1 PRDX2 PTEN SOD2 SOX17 SOX2 SRC STAT1 STAT2 TGFB1 TIM3 TIMP2 TLR4 TNF TNFRSF10A TNFRSF10B TNFRSF11B TNFRSF1A TP53 TWIST1 VEGFA VIM

RNA (40): BRAF BRCA1 CASP3 CAT CCNA2 CCNB1 CCND1 CDH1 CSTB CTSB CXCL8 CYP1B1 EGFR EPCAM ERBB2 ERBB3 ERBB4 F3 FAS GAPDH GATA3 GSTM4 GSTP1 HIST1H2AC JUN MDM2 MET MKI67 MYC NANOG PDGFRA PTEN SOD2 TFRC TGFB1 TIMP2 TNFRSF10A TNFRSF10B TP53 TRAPPC2

Overlap (31): BRAF BRCA1 CASP3 CAT CCNA2 CCNB1 CDH1 CSTB CTSB CXCL8 EGFR EPCAM ERBB2 ERBB3 ERBB4 F3 FAS JUN MKI67 MDM2 MET MYC NANOG PDGFRA PTEN SOD2 TGFB1 TIMP2 TNFRSF10A TNFRSF10B TP53

Supplementary table 2. Top 10 genes identified by PCA using either proteins or mRNAs of 31 common genes that contribute the most to the differentiation of A549, SKBR3 and K562 cells.

Gene_ID	Rank_Protein_PCA	Score (PC1-3) Protein_PCA	Rank_RNA_PCA	Score (PC1-3) RNA_PCA
CCNB1	1	0.509	4	0.384
EGFR	2	0.477	10	0.286
CCNA2	3	0.437	5	0.355
FAS	4	0.430	NA	NA
ERBB2	5	0.407	3	0.399
CXCL8	6	0.385	NA	NA
MET	7	0.364	1	0.444
TP53	8	0.327	7	0.348
MKI67	9	0.298	6	0.349
EPCAM	10	0.286	9	0.294
TGFB1	NA	NA	2	0.421
ERBB3	NA	NA	8	0.344

Note: NA means not available.

Supplementary table 3. Primer and probe sequences for qPCR of mRNA assays

Gene Name	Primary Forward Primer	Primary Reverse Primer	probe_sequence
BRAF	GACGGGACTCGAGTGATGAT	CACATCACCATGCCACTTTC	AGATTCCTGATGGGCAGATTACAGTGG
BRCA1	ACAGCTGTGTGGTGCTTCTGTG	CATTGTCCTCTGTCCAGGCATC	CATCATTCACCCTTGGCACAGGTGT
CASP3	TTTTTCAGAGGGGATCGTTG	CGGCCTCCACTGGTATTTTA	TTTCATTATTCAGGCCTGCCGTGGTAC
CAT	CTGGAGAAGTGCGGAGATTCA	GTTCCTCATTCAGCACGTTCAC	CTGCCAATGATGATAACGTTACTCAGG
CCNA2	CTGGAAAGTCTTAAGCCTTGTCTCA	GTTGAGGAGAGAAACACCATGATACT	CACCAGACCTACCTCAAAGCACCACAGCA
CCNB1	GCTGATCCAAACCTTTGTAGTGAA	ACTTCCCGACCCAGTAGGTA	TGACTGCTTGCTCTTCCTCAAGTTGTCT
CCND1	AGAGGCGGAGGAGAACAAA	AGGGCGGATTGGAAATGAAC	ACATCTGTGGCACAGAGGGCAACGA
CDH1	TGAGTGTCCCCCGGTATCTTC	CAGCCGCTTTCAGATTTTCAT	CCTGCCAATCCCGATGAAATTGGAAAT
CSTB	TGTCATTCAAGAGCCAGGTG	GAACACTCGCAGGTGTACGA	ACAAACTACTTCATCAAGGTGCACGTCG
CTSB	CTGGTTTCTGGTGGCCTCTA	CGTTGACGTGGTGCTCAC	CCATGTAGGGTGCAGACCGTACTCCA
CXCL8	ACACTGCGCCAACACAGAAA	CCCTCTGCACCCAGTTTTCC	CTGATGGAAGAGAGCTCTGTCTGGAC
CYP1B1	TGGCTGCTCCTCCTCTTCA	GGGCTGGTCACCCATACAA	CTCGAGTGCAGGCAGAATTGGATCAG
EGFR	ACGTACCAGATGGATGTGAACC	CACGAGCCGTGATCTGTCA	AGGGCAAATACAGCTTTGGTGCCACCT
EPCAM	GCTGGTGTGTGAACACTGCT	ACGCGTTGTGATCTCCTTCT	AAGTTTGCGGACTGCACTTCAGAAGGA
ERBB2	CCGGGAGTTGGTGTCTGAA	CGGTAGAAGGTGCTGTCCAA	CTTTGTGGTCATCCAGAATGAGGACTTGG
ERBB3	GTGAGGTGGTGATGGGGAA	ACGAGGACATAGCCTGTCAC	ACACAATGCCGACCTCTCCTTCCTGC
ERBB4	GAGGAGAGCTGGATGAGGAA	CCGAGAAACAAAAGGGTTCTCC	CATGACTCCTATGCGAGACAAACCCAAAC
F3	TCCCCAGAGTTCACACCTTAC	CACTTTTGTTCCCACCTGTTCA	TGGAGACAAACCTCGGACAGCCAAC
FAS	AGAAGGGAAGGAGTACACAGAC	CCGGGTGCAGTTTATTTCCA	CAGAAGATGTAGATTGTGTGATGAAGGAC
GAPDH	GCCAAGGTCATCCATGACAA	CATCACGCCACAGTTTCCC	CCACAGTCCATGCCATCACTGCCAC
GATA3	CAGAACCGGCCCCTCATTAA	CCATTGGCATTCCTCCTCCA	CGTCCTGTGCGAACTGTCAGACCACC
GSTM4	CAGGCTATGGACGTCTCCAA	TTCCTCCAAGTATTCTGGCTTCA	CAGAGTCTGCTACAGCCCTGACTTTG
GSTP1	AGAACCAGGGAGGCAAGAC	GGCTAGGACCTCATGGATCA	CCAGATCTCCTTCGCTGACTACAACC
HIST1H2AC	ACGACGAGGAGCTCAACAAA	AAGCACGGCCTGGATGTTA	TGACCATTGCTCAGGGCGGCGTCC
JUN	TCCACGGCCAACATGCT	CCACTGTTAACGTGGTTCATGAC	AGGGAACAGGTGGCACAGCTTAACAGA
MDM2	CTACAGGGACGCCATCGAAT	TGAATCCTGATCCAACCAATCA	CGGATCTTGATGCTGGTGTAAGTGAACATTC
MET	TGGACAATGATGGCAAGAAA	GATGATTCCCTCGGTCAGAA	CACTGTGCTGTGAAATCCTTGAACAGA
MKI67	GCTCGACCCTACAGAGTGCT	CTCCTTCACTGGGGTCTTGA	ACTTCATTTCCAACCAAAAAATGGACTTTAAGGA
MYC	TGAGGAGACACCGCCCAC	CAACATCGATTTCTTCCTCATCTTC	CCAGCAGCGACTCTGAGGAGGAACA
NANOG	ATACCTCAGCCTCCAGCAGA	TCTGGAACCAGGTCTTCACC	AGAACTCTCCAACATCCTGAACCTCAGC
PDGFRA	GGGAGTTTCCAAGAGATGGA	CTTCAACCACCTTCCCAAAC	AGTGCTTGGTCGGGTCTTGG
PTEN	AAGACATTATGACACCGCCAAAT	ATGATTGTCATCTTCACTTAGCCATT	TGCAGAGTTGCACAATATCCTTTTGAAGACC
SOD2	AGGAACGGGGACACTTACAA	TCAATCCCCAGCAGTGGAATA	CTGCTTGTCCAAATCAGGATCCACTG
TFRC	AAAATCCGGTGTAGGCACAG	CACCAACCGATCCAAAGTCT	AGCATTATCTTTGCCAGTTGGAGTGC
TGFB1	CCCTGGACACCAACTATTGC	GTCCTTGCGGAAGTCAATGT	CTCCACGGAGAAGAACTGCTGCGTG
TIMP2	ACCCAGAAGAAGAGCCTGAA	GGAGATGTAGCACGGGATCA	CAGGTACCAGATGGGCTGCGAGTG
TNFRSF10A	TTCCAGCAAATGGTGCTGAC	GCTGGTCCCAGGAGTCAAA	CACTGAGACTCTGATGCTGTTCTTTGAC
TNFRSF10B	TCCGAAAGTGCTGGGATTAC	CCCCCAATTATTTCATGTCG	AGCCACCAGCCAGGCCAAGCTATT
TP53	TAACAGTTCCTGCATGGGCGGC	AGGACAGGCACAAACACGCACC	CGGAGGCCCATCCTCACCATCATCA
TRAPPC2	CTCGACCTCGTAGATGAGAACA	ACAAATGCCGACACAAACCA	CGAACAACATGTACTTGAAAACTGTGGAC

Supplementary Fig. 1

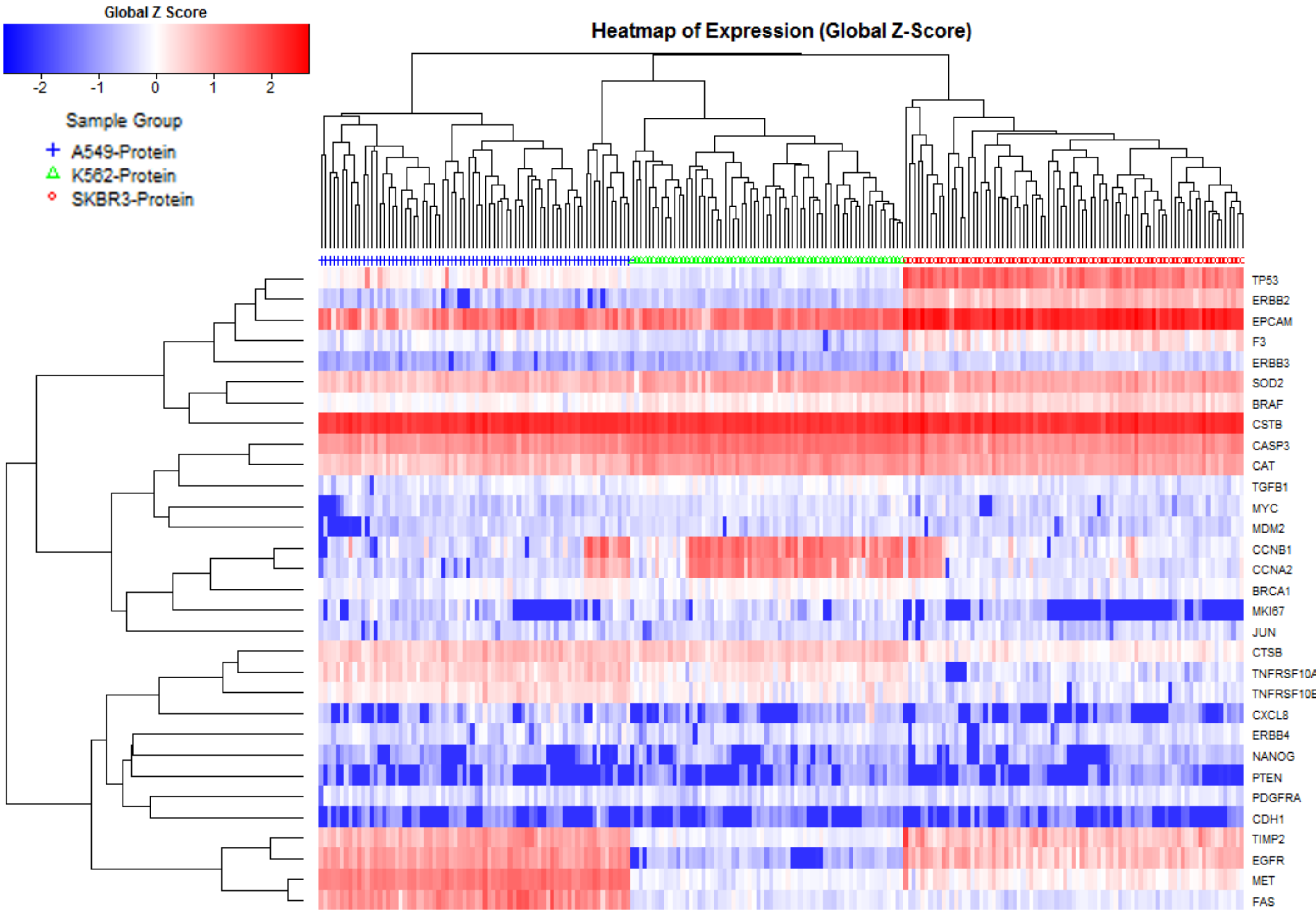

Supplementary Figure 1. Hierarchical clustering analysis of A549, SKBR3 and K562 single cells based on protein levels of 31 genes.

Supplementary Fig. 2

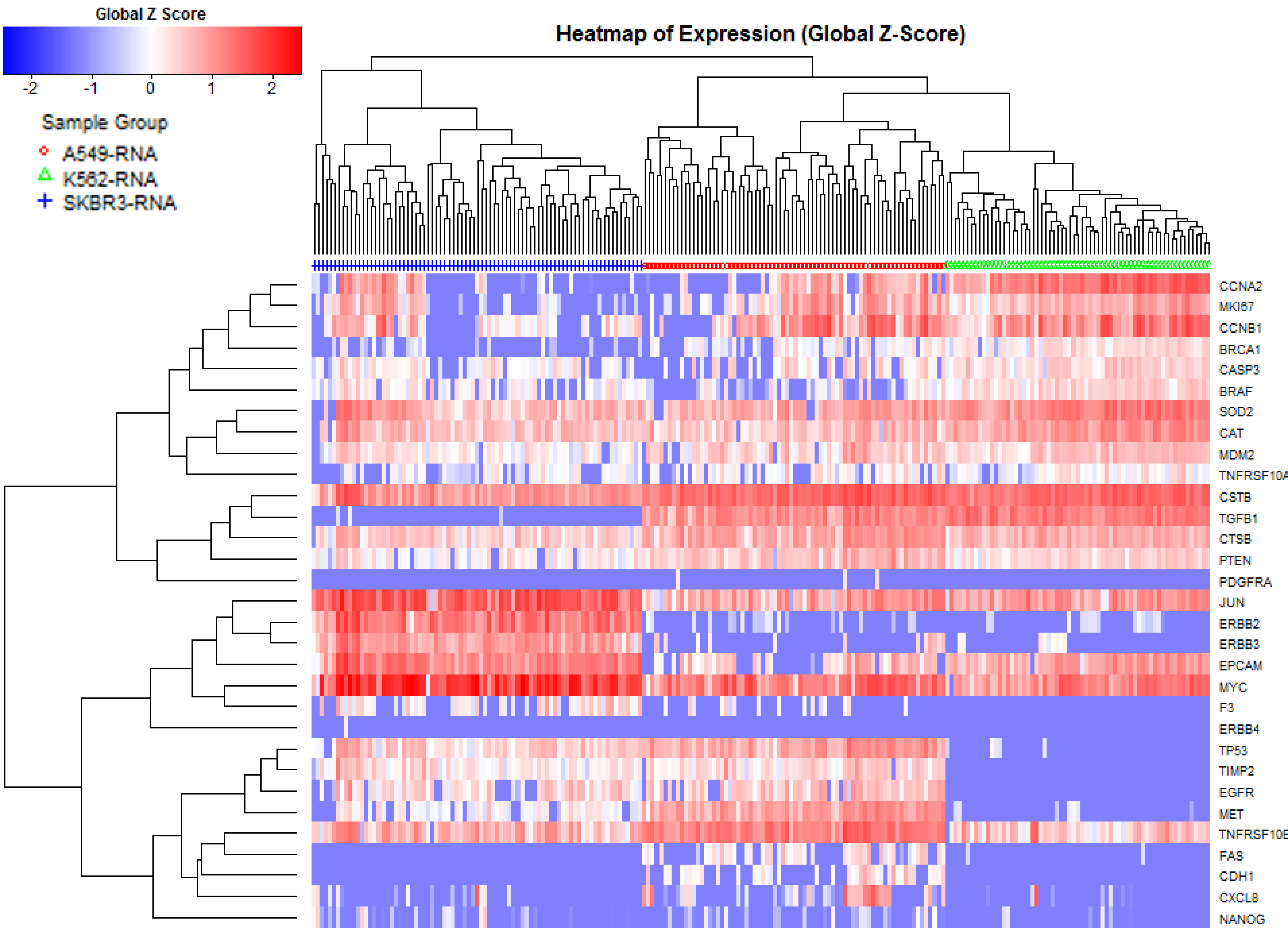

Supplementary Figure 2. Hierarchical clustering analysis of A549, SKBR3 and K562 single cells based on mRNA levels of 31 genes.

Supplementary Fig. 3

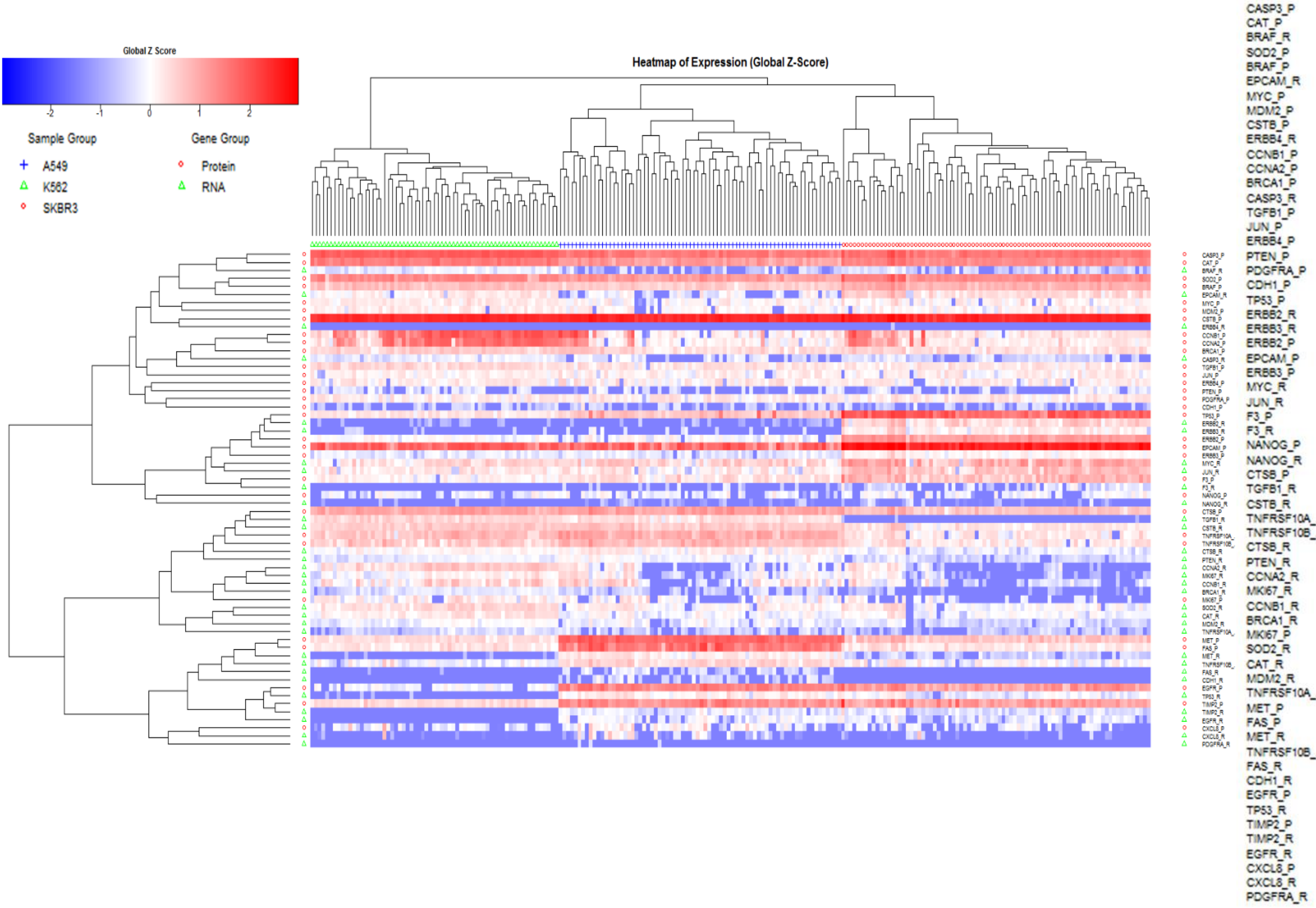

Supplementary Figure 3. Hierarchical clustering analysis of A549, SKBR3 and K562 single cells based on both protein and mRNA levels of 31 genes.

Supplementary Fig. 4

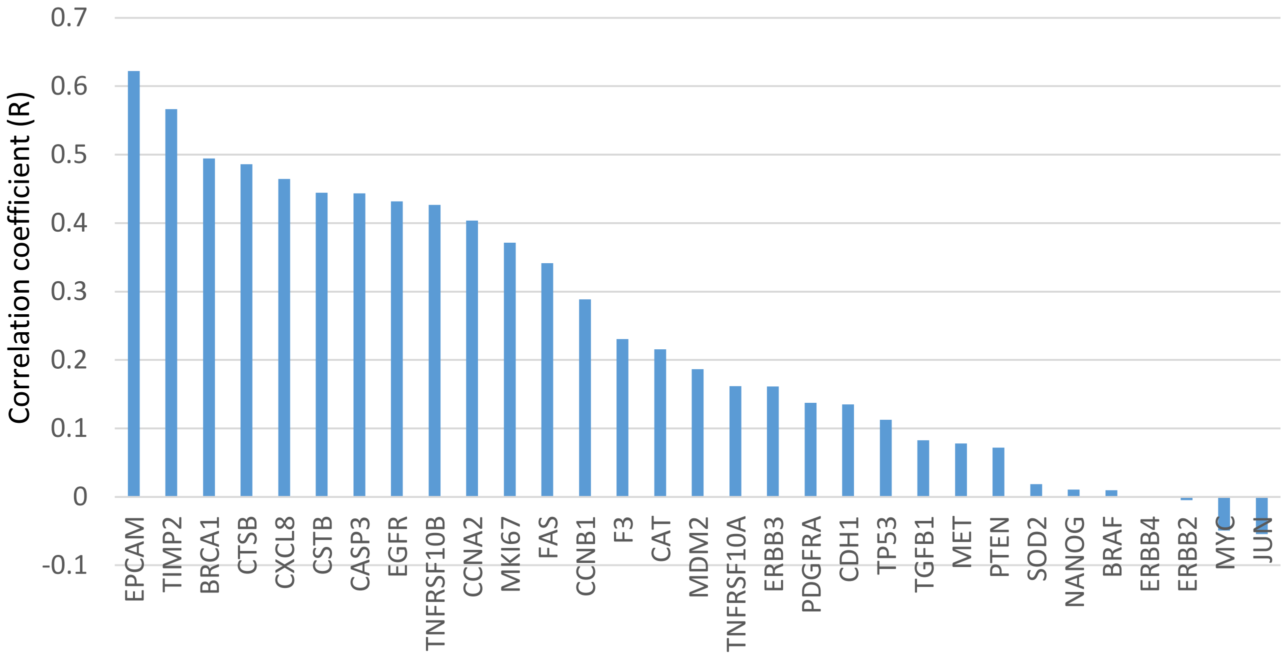

Supplementary Figure 4. Correlation between protein and mRNA levels of individual genes in A549 single cells.

Supplementary Fig. 5

(A)

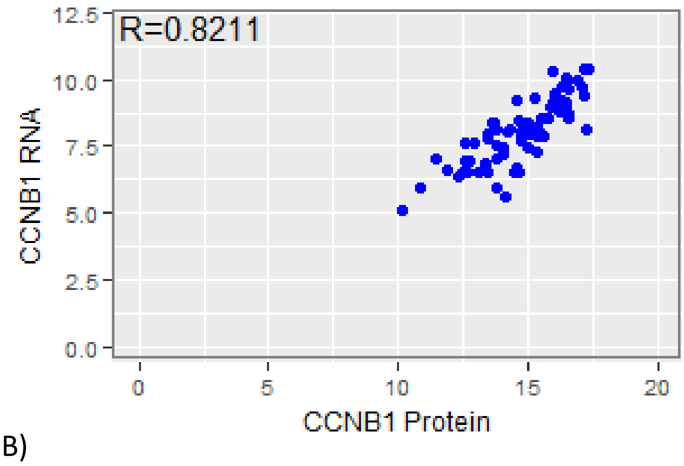

(B)

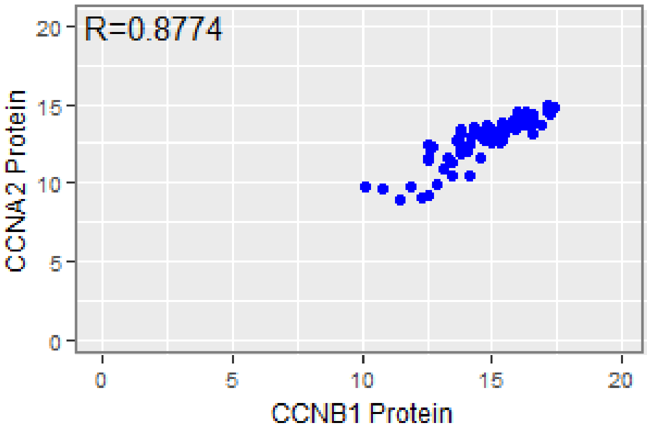

(C)

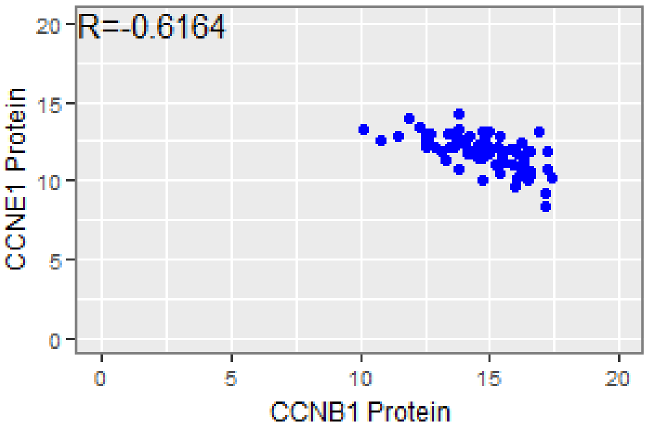

Supplementary Figure 5. Correlation analysis of the A549 cell subpopulation highlighted with blue color in Fig. 5F. (A) Correlation between CCNB1 protein and RNA. (B) Correlation between CCNB1 protein and CCNA2 protein. (C) Correlation between CCNB1 protein and CCNE1 protein.

Supplementary Fig. 6

(A)

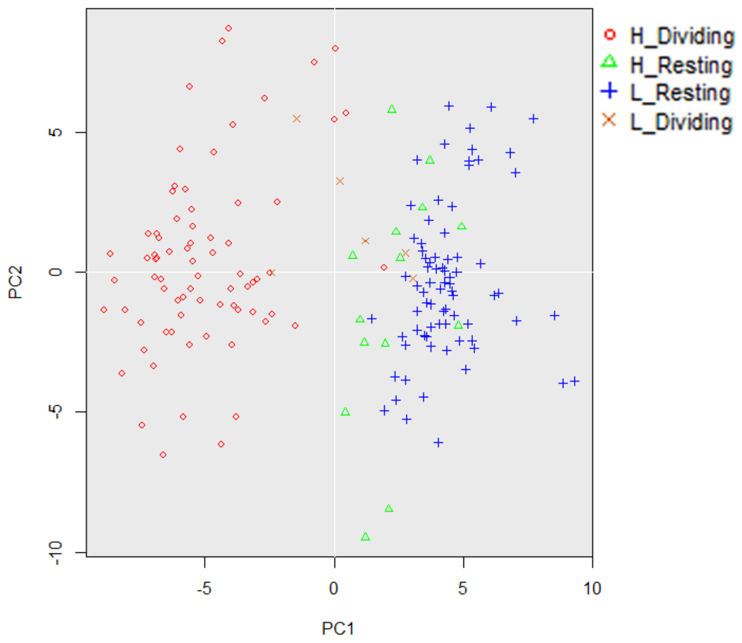

(B)

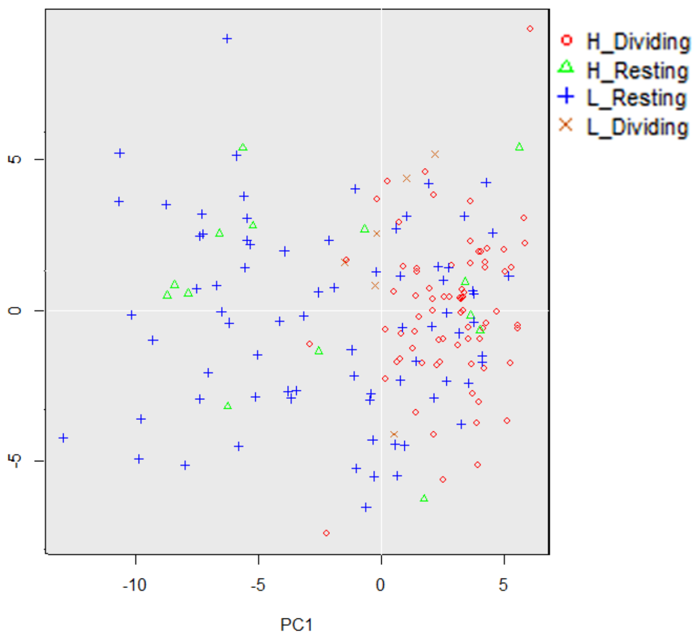

(C)

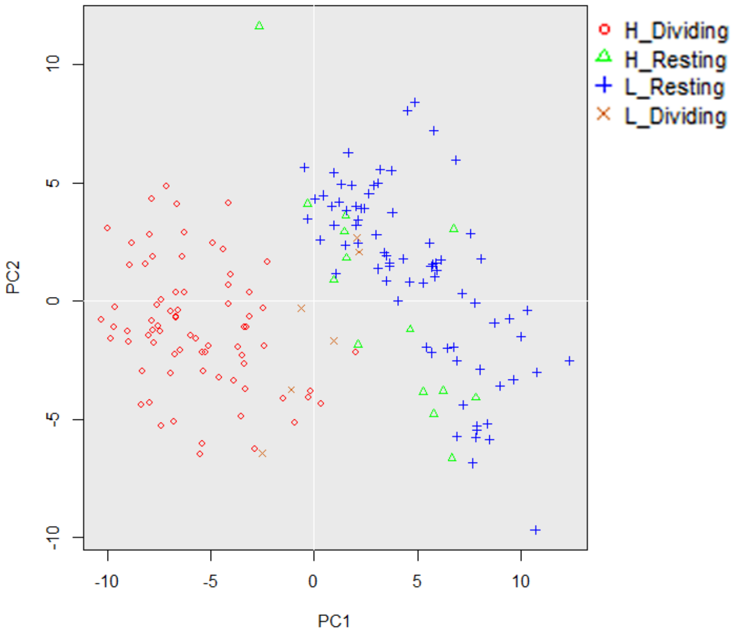

Supplementary Figure 6. Principal component analysis (PCA) of A549 single cells sorted by DNA content (H-high DNA content; L-low DNA content). Dividing and resting cell subpopulations were assigned based on the correlation between CCNB1 protein and mRNA (see Fig. 5F,G). The analysis was conducted using either protein levels (A), mRNA levels (B), or both protein and mRNA (C) of 31 genes.

Supplementary Fig. 7

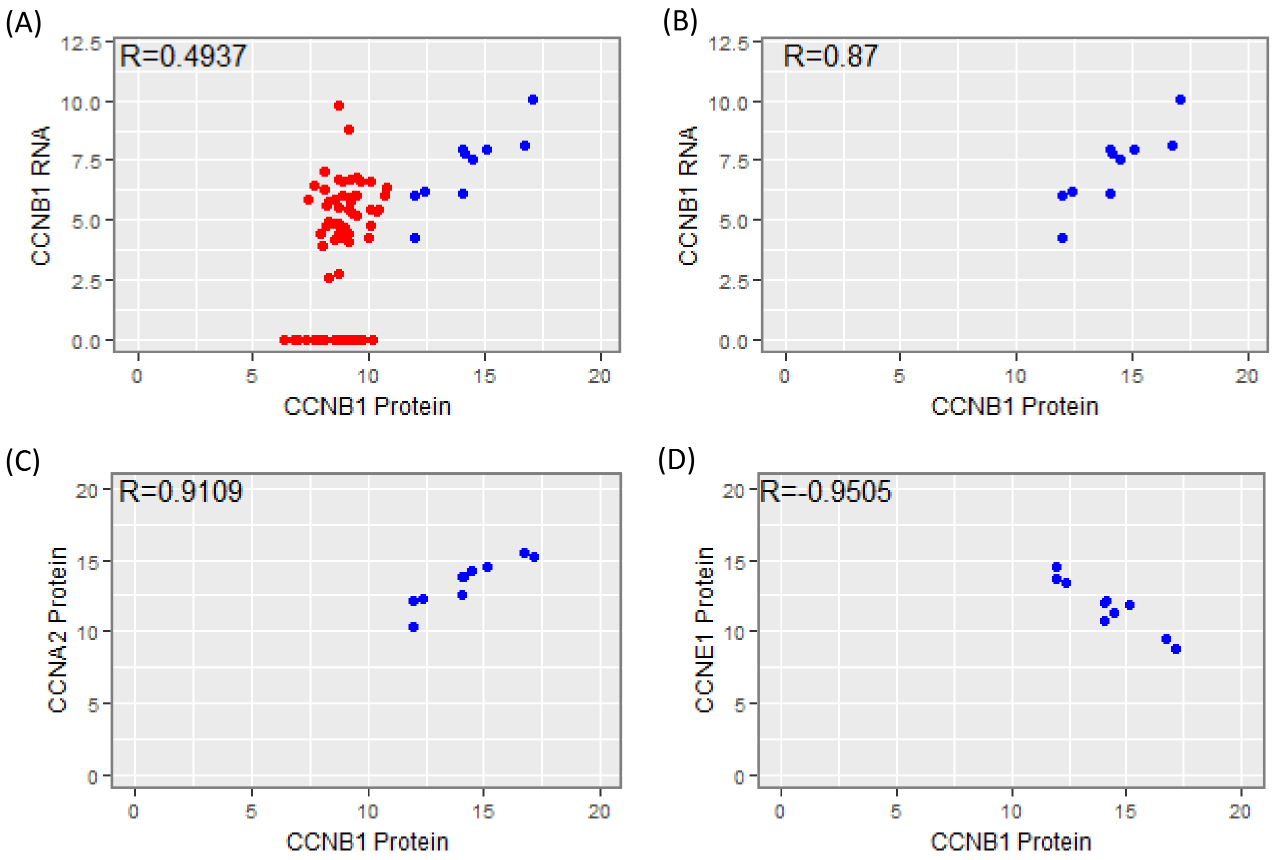

Supplementary Figure 7. Correlation analysis of proteins and mRNAs in SKBR3 single cells. (A) Correlation between CCNB1 protein and mRNA in the whole population of cells. (B) Correlation between CCNB1 protein and RNA in the subpopulation highlighted with blue color in (A). (C) Correlation between CCNB1 protein and CCNA2 protein in the same subpopulation as in (B). (D) Correlation between CCNB1 protein and CCNE1 protein in the same subpopulation as in (B).
